# Editorial: Underrepresentation of Women in Science: International and Cross-Disciplinary Evidence and Debate

**DOI:** 10.3389/fpsyg.2017.02352

**Published:** 2018-01-22

**Authors:** Wendy M. Williams

**Affiliations:** Department of Human Development, Cornell University, Ithaca, NY, United States

**Keywords:** women in science, underrepresentation of women, women in STEM, STEM careers, work-life balance

There is no shortage of articles and books exploring women's underrepresentation in science. Everyone is interested—academics, politicians, parents, high school girls (and boys), women in search of college majors, administrators working to accommodate women's educational interests; the list goes on. But one thing often missing is an evidence-based examination of the problem, uninfluenced by personal opinions, accounts of “lived experiences,” anecdotes, and the always-encroaching inputs of popular culture. This is why this special issue of *Frontiers in Psychology* can make a difference. In it, a diverse group of authors and researchers with even more diverse viewpoints find themselves united by their empirical, objective approaches to understanding women's underrepresentation in science today.

## Overview of articles in special issue

The questions considered within this special issue span academic disciplines, methods, levels of analysis, and nature of analysis; what these article share is their scholarly, evidence-based approach to understanding a key issue of our time.

### Sexism in professorial hiring

Ceci and Williams re-visited the experimental paradigm from their 2015 *Proceedings of the National Academy of Sciences* article in which (in four of their five experiments) faculty were asked to rate three short-listed finalists for a tenure-track position. The 2015 study revealed a 2:1 preference for women when finalists were equivalently excellent. The new study contrasted a male finalist who was slightly superior to the female finalist. Women's advantage vanished when the male applicant was depicted as slightly stronger, suggesting that fears that affirmative action goals will undermine hiring of most-qualified applicants are unfounded.

Allen-Hermanson examined an overlooked aspect of the women's underrepresentation debate: Are philosophers prejudiced against hiring women applicants despite professing conscious, explicit egalitarian beliefs? Unlike other humanities departments, philosophy departments have far fewer women professors than might be expected. He reviewed several recent data sets demonstrating that female applicants are favored when it comes to tenure-track hiring in philosophy departments.

### Exploring the gender gap via national datasets

Using 1993–2010 nationally representative data, Kahn and Ginther examined whether the gender gap in engineering has narrowed recently. They discovered that the majority of the gender retention gap was due to women leaving the labor force coincident with child-bearing. There was no gender retention difference by 7–8 years post-bachelors for those full-time employed; single childless women were more likely than men to remain in engineering than were single childless men, and women who left engineering entirely were just as likely as men who left to remain in math-intensive fields. Their findings caution against past assertions that women do not persist in STEM fields as long as men.

In their latest meta-analysis, Su and Rounds examined data from 52 samples entailing over 430,000 respondents between 1964 and 2007. Gender differences in interests favoring males were largest in engineering-related fields, and favored women in allied health fields and social sciences. This adds to the large body of empirical findings that have revealed similar sex differences along the people-thing dimension.

Miller and Wai reported the results of their analysis of longitudinal data to examine the baccalaureate-to-PhD transition. In contrast to the traditional leaky pipeline metaphor, they found that over time, women have segued from the baccalaureate to PhD programs in increasing numbers. Their work suggests that researchers and policy makers need to look elsewhere for causes of women's underrepresentation.

Wang et al. studied factors predicting gender differences in selection of STEM occupations, and whether math task values and altruism mediate the pathway through which gender affects STEM career choice through math achievement. Based on longitudinal analyses, they found that the association between gender and working in a STEM career by one's early- to mid-thirties was mediated by math achievement scores in twelfth grade; females did more poorly on standardized math tests than did males.

### Stereotypes about “brilliance” and “male-oriented” fields

Meyer et al. examined field-specific beliefs regarding the importance of brilliance. They provide support for the hypothesis that women are most likely to be underrepresented in fields that members believe require raw intellectual talent, which women are stereotyped to possess less of than do men. The beliefs of participants with exposure to a field predicted the magnitude of the field's gender gap, independent of their beliefs about the level of mathematical ability required. Their findings are consistent with female high school students taking fewer AP courses in all areas of science except biology (Ceci et al., 2014).

Cheryan et al. presented data and argument showing that modern American culture stereotypes as male-oriented those fields that involve social isolation, an intense focus on machinery, and inborn brilliance. These stereotypes are compatible with qualities that are typically more valued in men than women in American culture. Their work continues their recent insights and is consistent with the findings of some of the other contributors, particularly Meyer et al.

Smyth and Nosek explored whether variation in female representation across scientific disciplines is associated with differences in the strength of gender-science stereotypes, explicit and implicit, held by men and women in these fields. For explicit stereotypes that associate science with “male,” the strength of stereotyping varied across scientific disciplines as a function of gender ratios in the disciplines; however, implicit stereotypes did not vary as a function of such ratios. Giving currency to their findings is recent evidence that children continue to associate science with being male (Miller et al., in press).

### Importance of competitive schools and perceived math ability

Mann et al. analyzed findings from PISA data from 55 countries. They placed schools along a continuum from most to least competitive, based on average math and science performance. Schools that are most competitive are often associated with stronger math-science environments. The authors found that the aspirations gender gap narrowed for high-performing students in stronger performance environments.

Nix et al. reported an analysis of longitudinal, nationally representative high school data. They found that perceived mathematics ability when under challenge predicted important outcomes such as taking advanced science courses in high school, and that high school men scored higher than women did in their perceived ability under mathematics challenge. Their findings are consistent with female high school students taking fewer AP courses in all areas of science except biology (Ceci et al., 2014).

### Wisdom from the trenches of academia

Finally, Williams et al. collected and analyzed an original national empirical dataset in which provosts, deans, associate deans, and department chairs of STEM fields at 96 U.S. research-intensive universities rated the quality and feasibility of strategies for retaining women in STEM fields. For example, administrators agreed that gender quotas were a weak idea, and that campus childcare centers were an excellent idea. Women administrators were more supportive than were men of shared tenure lines, and saw it as more feasible for men to stop the tenure clock for 1 year for childrearing.

In sum, readers will find multiple perspectives in this special issue, and the editors hope it will stimulate new directions of thinking and scholarship on women and science.

## Author contributions

The author confirms being the sole contributor of this work and approved it for publication.

### Conflict of interest statement

The author declares that the research was conducted in the absence of any commercial or financial relationships that could be construed as a potential conflict of interest.
